# Primary lung adenocarcinoma with gross calcification and suspected bone formation

**DOI:** 10.20407/fmj.2019-019

**Published:** 2020-02-11

**Authors:** Atushi Kato, Masamichi Hayashi, Disuke Totii, Yosuke Sakakibara, Shingo Maeda, Kazuyoshi Imaizumi

**Affiliations:** 1 Department of Respiratory Medicine, Fujita Health University, School of Medicine, Toyoake, Aichi, Japan; 2 Department of Respiratory Surgery, Fujita Health University, School of Medicine, Toyoake, Aichi, Japan

**Keywords:** Gross calcification, Bone formation, Primary lung adenocarcinoma, Differential diagnosis

## Abstract

Calcification in a lung tumor suggests that it is a benign tumor such as a hamartoma or a sclerosing lung cell tumor. In contrast, carcinoid, lung cancer, carcinosarcoma, and sarcoma rarely harbor calcification. Primary lung adenocarcinomas with gross calcification that is suggestive of bone formation are very rare. It is difficult to distinguish between calcification and bone formation purely on the basis of image definitive diagnosis of bone formation being difficult in the absence of a large surgical specimen. Lung cancers with bone formation are exceedingly rare: to the best of our knowledge, only 13 cases have been reported. Careful attention is needed when differentiating between benign and malignant tumors. Here, we report a case of primary lung adenocarcinoma with gross calcification that was suggestive of bone formation.

## Introduction

Calcification in a lung tumor suggests that it is a benign tumor such as a hamartoma or a sclerosing lung cell tumor.^1^ Although carcinoid,^1^ lung cancer,^2–4^ carcinosarcoma,^5^ and sarcoma^6^ rarely harbor calcification, a malignant tumor should be included in the differential diagnosis. Moreover, it is very rare to find gross calcification that is suggestive of bone formation in lung cancers. Because it is difficult to differentiate between calcification and bone formation purely on the basis of image, a definitive diagnosis of bone formation cannot be made without obtaining a large surgical specimen.

## Case report

A 44-year-old man was referred to our hospital because of an abnormal shadow on a chest radiograph. Chest computed tomography (CT) four months later showed a 4 cm diameter tumor with a coarse high-density area in the right S10.

Laboratory tests showed high carcinoembryonic antigen (CEA) (62.7 ng/mL) and triglyceride (TG) (220 mg/dL) concentrations level. Thyroid and parathyroid function were normal (Table 1).

**Figure 1:**
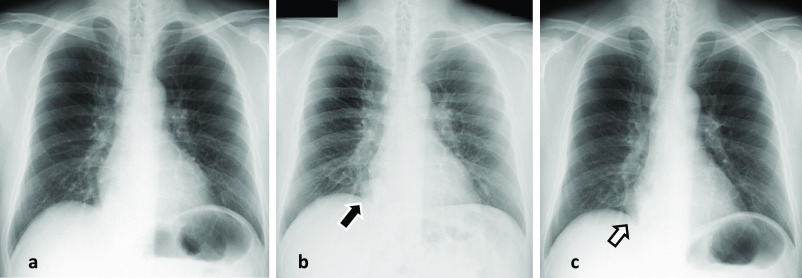
Chest X-ray a. taken 2 years before presentation is normal. b. taken 1 year before presentation shows a coin lesion in the right lower lung field. (black arrow) c. taken 4 months before referral to our institution shows a nodular lesion has increased in size. (white arrow)

A chest radiograph taken 2 years before presentation was normal, whereas a coin-like nodular shadow was detected in the lower right lobe one year before presentation to our institution. That nodule gradually increased in size and was recognized as a significant mass four months before referral to our institution (Figure 1).

**Figure 2:**
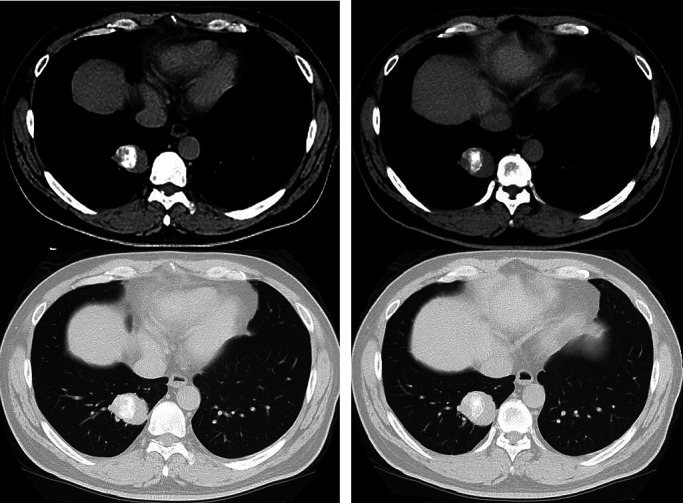
Chest CT showed a mass lesion, 4 cm in diameter, in the right lower lobe S10 with coarse high density area (gross calcification) and pleural indentation.

Chest CT showed a 4 cm diameter mass with irregular edges, pleural indentation, and gross calcification in the right S10. The fat component of the tumor was unclear. The mediastinal and hilar lymph nodes were not enlarged (Figure 2).

The tumor was suspected of being a lung hamartoma. However, because the tumor was seen to partially project into the bronchus on review of the chest CT images and the concentration of the tumor marker CEA was high, it was considered that the possibility of a malignant tumor warranted a bronchoscopy to establish a definitive diagnosis.

**Figure 3:**
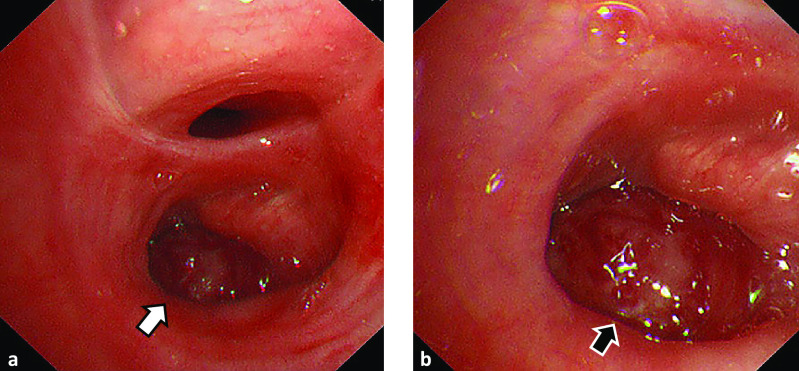
Bronchoscopic findings a. Tumor like a polyp with not smooth surface occludes in right lower bronchus. (white arrow) b. Enlarged view of Figure 3a. (black arrow)

Bronchoscopy revealed a polyp-like tumor with an irregular surface protruding into and occluding the entrance of the right lower bronchus. A biopsy was taken from the tumor (Figure 3).

**Figure 4:**
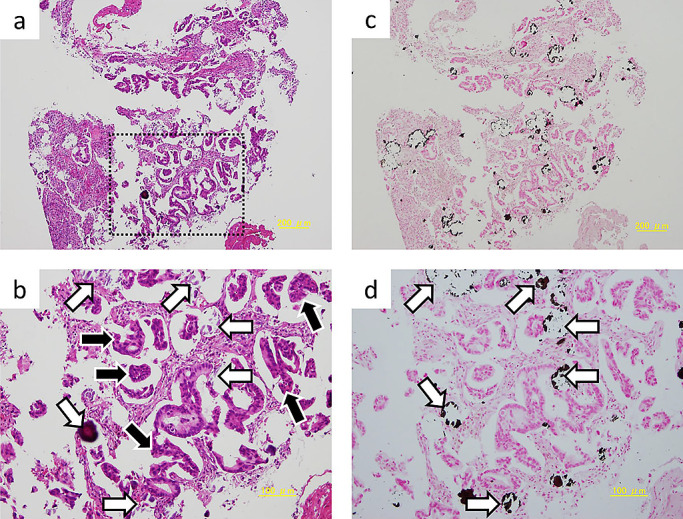
Pathological findings showed irregularly atypical ductal hyperplasia (black arrow) with small calcification (white arrow) in the inside of tumor. a. Magnification 40× (H.E. stain), b. Magnification 100× (H.E. stain), c. Magnification 40× (Von Kossa stain), d. Magnification 100× (Von Kossa stain)

Pathological examination of the biopsy specimen revealed irregular ductal hyperplasia with atypical cells, and many small areas of calcification, some of which were within the tumor. Von Kossa staining confirmed calcium deposition. Immunostaining was positive for CK7, TTF-1 and Napsin A, and negative for CK20. Thus, the pathological diagnosis was primary lung adenocarcinoma with remarkable calcification (Figure 4).

**Figure 5:**
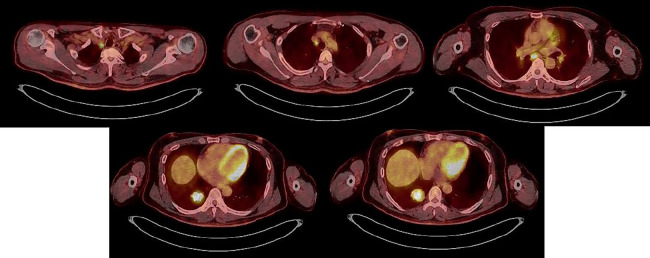
PET-CT showed the accumulation for a mass lesion (SUV max 12.64) in the right lower lobe S10 and lymph nodes #1R (SUV max 13.15), #4R (SUV max 5.52), #7 (SUV max 8.97).

Fluorodeoxyglucose-positron emission tomography (FDG-PET)/CT showed accumulation in a mass lesion with SUV max 12.64 in the right lower lobe S10. Accumulation in lymph nodes was as follows: #1R (SUV max 13.15), #4R (SUV max 5.52), #7 (SUV max 8.97) (Figure 5).

Brain MRI showed no significant occupying lesion.

Although FDG-PET/CT showed accumulation in the mediastinal lymph nodes, a thoracic surgeon considered that lymph node dissection could be performed via a right thoracic approach. However, because pleural dissemination was noted intraoperatively, only an exploratory thoracotomy was performed. The postoperative diagnosis was pT2aN3M1a, stage IV. The patient is currently undergoing chemotherapy.

The authors obtained the patient’s consent for publication of details of his case.

## Discussion

Calcification detected by image, which may be central, layered, hole, diffuse, or popcorn-like, strongly suggests that a nodular shadow in the lung is benign.^7^

Gross calcification is usually associated with benign lesions such as ectopic calcification due to old inflammation and bone or cartilage formation. However, calcification or bone formation occurs in lung cancer or sarcoma.

Mahoney et al.^2^ reported that chest CT showed calcification in 5.7% of 353 patients with lung cancer. Grewal et al.^3^ reported detecting calcification in 10.6% of 500 patients. Okimoto et al.^4^ reported detecting calcification in 5.6% of 320 patients with tumors of confirmed histological types. These findings suggest that 5% to 10% of lung cancers are associated with calcification.

When a lung nodule or mass shadow is accompanied by gross calcification, care should be taken to distinguish between benign and malignant lesions. As in the present case, when high concentrations of tumor markers or imaging findings suggestive of malignancy are observed, it is necessary to make a definitive diagnosis by performing examinations like a bronchoscopy rather than merely following the patient up.

Unfortunately, our patient’s tumor could not be removed to determine whether the gross calcification seen on image represented bone formation. It is difficult to distinguish between calcification and bone formation purely on the basis of imaging studies; thus, definitive diagnosis of bone formation is difficult in the absence of a large surgical specimen. Lung cancer with bone formation is exceedingly rare: to the best of our knowledge, only 13 cases^8–12^ have been reported. These patients comprised nine men and four women of average age 63 years. The histologic types were one squamous cell carcinoma, one adenosquamous cell carcinoma, and 11 adenocarcinomas. Kuribayashi et al. reviewed 2269 surgically resected primary lung carcinomas and identified 33 (about 1.5%) with heterotopic ossification, including 15 with intratumoral heterotopic ossification and 18 with extratumoral heterotopic ossification. All cases with intratumoral heterotopic ossification were adenocarcinomas.^13^

Lung cancers with bone formation are believed to progress slowly and reportedly have a good prognosis if diagnosed at an early stage.^14^ However, our patient’s tumor progressed quickly; thus, the prognosis of lung cancer with gross calcification is not necessarily good. More cases need to be accumulated.

## Figures and Tables

**Table1:** Laboratory data

Hematology		Biochemistry		Serology	
WBC	5,400/μl	T-Bil	0.7 mg/dl	CRP	0.3>mg/l
Neutrophils	70%	AST	20 IU/L		
Eosinophils	2%	ALT	20 IU/L	Tumor marker	
Lymphocytes	24%	LDH	169 IU/L	CEA	62.7 ng/ml
Monoctes	4%	ALP	201 IU/L	CYFRA	1.8 ng/ml
RBC	525×10^4^/μl	TP	6.8 g/dl	ProGRP	46.2 pg/ml
Hb	15.0 g/dl	Alb	4.4 g/dl	IL-2R	390 U/ml
Hct	44.6%	BUN	12.5 mg/dl		
Plt	25.6×10^4^/μl	Cre	0.85 mg/dl	T-SPOT	negative
		Na	141 mEq/L		
		K	4.1 mEq/L		
		Cl	105 mEq/L		
		Ca	9.2 mg/dl		
		FBS	102 mg/dl		
		HbA1c (NGSP)	5.6%		
		Total cholesterol	215 mg/dl		
		Triglyceride (TG)	220 mg/dl		
